# An inducible mouse model of podocin-mutation-related nephrotic syndrome

**DOI:** 10.1371/journal.pone.0186574

**Published:** 2017-10-19

**Authors:** Mansoureh Tabatabaeifar, Tanja Wlodkowski, Ivana Simic, Helga Denc, Geraldine Mollet, Stefanie Weber, John Julius Moyers, Barbara Brühl, Michael Joseph Randles, Rachel Lennon, Corinne Antignac, Franz Schaefer

**Affiliations:** 1 Division of Pediatric Nephrology, Center for Pediatrics and Adolescent Medicine, University of Heidelberg, Heidelberg, Germany; 2 INSERM, U1163, Imagine Institute, Laboratory of Hereditary Kidney Diseases, Paris, France; 3 Paris Descartes-Sorbonne Paris Cité University, Paris, France; 4 Pediatric Nephrology, Center for Pediatrics and Adolescent Medicine, Philipps-University Marburg, Marburg, Germany; 5 Institute for Pathology, University of Heidelberg, Heidelberg, Germany; 6 Institute for Anatomy and Cell Biology, University of Heidelberg, Heidelberg, Germany; 7 Wellcome Trust Centre for Cell Matrix Research, University of Manchester, Manchester, United Kingdom; 8 Faculty of Biology Medicine and Health, University of Manchester, Manchester, United Kingdom; 9 Department of Genetics, Necker Hospital, Assistance Publique—Hôpitaux de Paris, Paris, France; University of Houston, UNITED STATES

## Abstract

Mutations in the *NPHS2* gene, encoding podocin, cause hereditary nephrotic syndrome. The most common podocin mutation, R138Q, is associated with early disease onset and rapid progression to end-stage renal disease. Knock-in mice carrying a R140Q mutation, the mouse analogue of human R138Q, show developmental arrest of podocytes and lethal renal failure at neonatal age. Here we created a conditional podocin knock-in model named *NPHS2* ^*R140Q/-*^, using a tamoxifen-inducible Cre recombinase, which permits to study the effects of the mutation in postnatal life. Within the first week of R140Q hemizygosity induction the animals developed proteinuria, which peaked after 4–5 weeks. Subsequently the animals developed progressive renal failure, with a median survival time of 12 (95% CI: 11–13) weeks. Foot process fusion was observed within one week, progressing to severe and global effacement in the course of the disease. The number of podocytes per glomerulus gradually diminished to 18% compared to healthy controls 12–16 weeks after induction. The fraction of segmentally sclerosed glomeruli was 25%, 85% and 97% at 2, 4 and 8 weeks, respectively. Severe tubulointerstitial fibrosis was present at later disease stage and was correlated quantitatively with the level of proteinuria at early disease stages. While R140Q podocin mRNA expression was elevated, protein abundance was reduced by more than 50% within one week following induction. Whereas miRNA21 expression persistently increased during the first 4 weeks, miRNA-193a expression peaked 2 weeks after induction. In conclusion, the inducible R140Q-podocin mouse model is an auspicious model of the most common genetic cause of human nephrotic syndrome, with a spontaneous disease course strongly reminiscent of the human disorder. This model constitutes a valuable tool to test the efficacy of novel pharmacological interventions aimed to improve podocyte function and viability and attenuate proteinuria, glomerulosclerosis and progressive renal failure.

## Introduction

Recessive mutations in the *NPHS2* gene, coding for the podocyte membrane protein podocin, constitute the most common genetic cause of childhood-onset nephrotic syndrome [1, 2]. Podocin is part of a multi-protein complex at the podocyte slit diaphragm that serves to recruit nephrin to the plasma membrane and link the slit diaphragm to the podocyte cytoskeleton [3–5].

Focal-segmental glomerulosclerosis (FSGS) is the typical histopathological finding of podocin-related glomerulopathy and progression to end-stage renal disease (ESRD) inevitably occurs, although the age at first disease manifestation and the rate of disease progression are rather variable. The phenotype is to a major degree related to the type and position of the mutations [6].

Among more than 120 pathogenic mutations described to date [7] the p.R138Q (c.413G>A) mutation is the most common, at least among Caucasians, representing 32% and 44% of all mutated alleles in two large European series [8, 9]. The resulting substitution of the highly conserved glutamine at this position for arginine leads to protein misfolding and retention in the endoplasmic reticulum (ER) [3, 10]. The R138Q mutation is associated with early disease onset and rapid progression to ESRD [9].

Several mouse models of podocin-related glomerulopathy have been developed over the past decade to elucidate the function of the protein, study the natural course of the disease and assess potential therapeutic approaches [10–12]. A constitutive podocin knockout (*Nphs2*^*-/-*^) mouse model revealed the requirement of podocin for the correct assembly of nephrin at the slit diaphragm. However, constitutive absence of podocin led to developmental arrest of podocytes and lethal renal failure at neonatal age [11]. Subsequently, to mimic the most common human genetic abnormality, a mouse strain carrying the R140Q mutation (knockin), the murine analogue of human R138Q, was generated [10]. Unfortunately, similar to the knockout model, the constitutive knock-in mice died from renal failure within the first 1–2 weeks of life. More recently, an inducible podocin knockout mouse model was generated using inducible Cre recombinase technology [12]. In these mice bearing one *Nphs2* allele with a floxed exon 2, one null *Nphs2* allele, and a podocyte-expressed, tamoxifen-responsive Cre recombinase transgene [13], podocin loss was induced by tamoxifen injection. When podocin deficiency is induced postnatally in mature kidneys, massive albuminuria develops which is followed by glomerulosclerosis and progressive renal failure, with death occurring at a median time of 11 weeks after induction of podocin deficiency [12]. While this phenotype resembles the course of podocin-related nephropathy in humans, the inducible podocin knockout model does still not allow one to study the fate of R140Q mutant podocin and mutation bearing podocytes *in vivo*. Therefore, the inducible podocin knockout is not a suitable model to explore innovative therapeutic approaches aimed to rescue R140Q mutant podocin function.

Here, we combined the previous approaches to generate a novel inducible knock-in mouse model of podocin-associated glomerulopathy. Cross-breeding of animals carrying one floxed podocin allele, one podocin allele bearing the R140Q mutation, and a tamoxifen-sensitive inducible Cre transgene enabled us to induce hemizygosity for the R140Q mutant, i.e. a genetic condition closely mimicking the human disease. We provide a detailed characterization of this potentially useful model of the most common single genetic abnormality causing human hereditary nephrotic syndrome.

## Results

### *Nphs2*^*R140Q/-*^ mice develop nephrotic syndrome and progressive kidney failure

**Fig 1:**
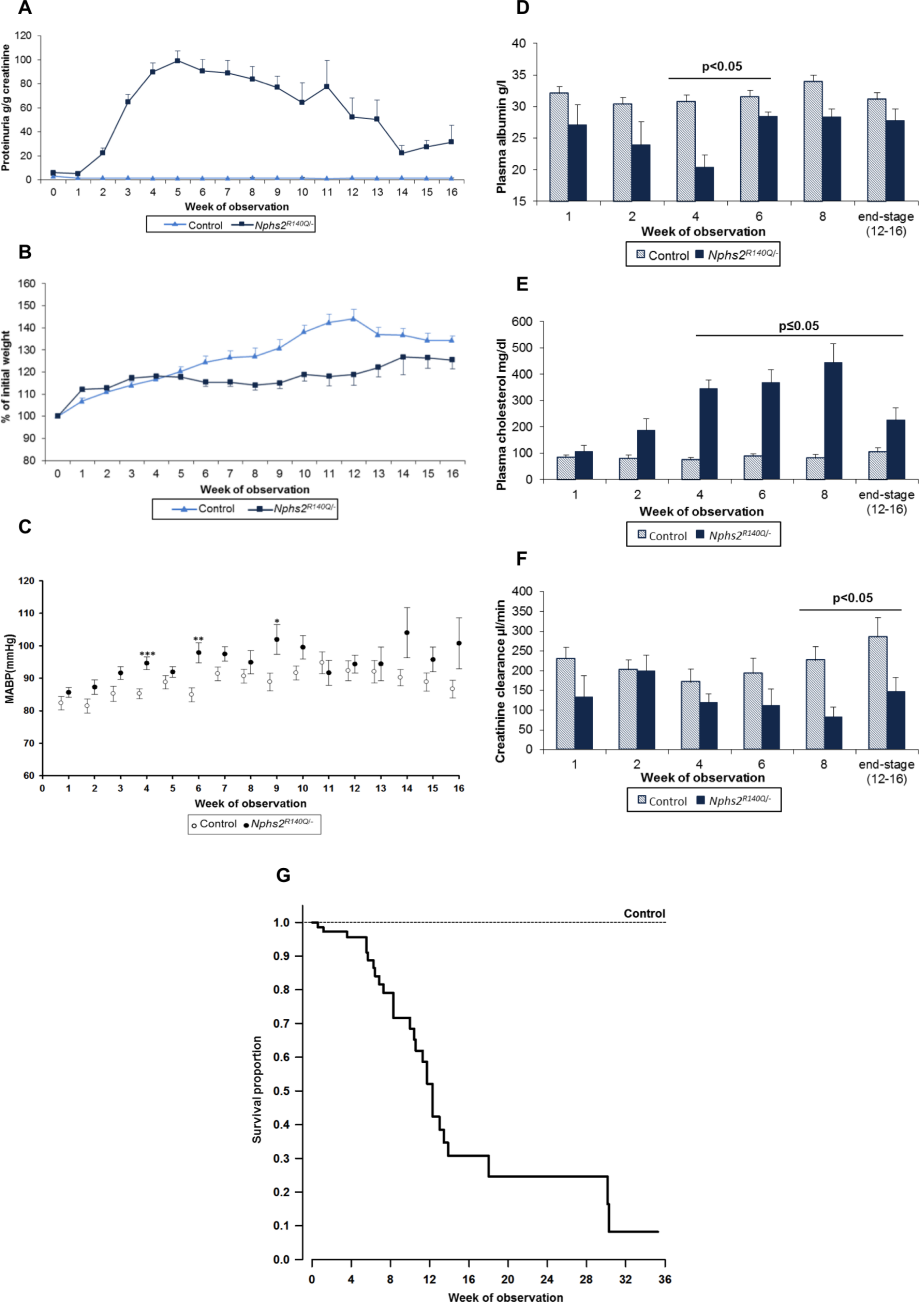
*Nphs2*^*R140Q/-*^ mice develop nephrotic syndrome. (A) Induced mice develop proteinuria (control n = 49; *Nphs2*^*R140Q/-*^ n = 57; p<0.0001), (B) display diminished weight gain (control n = 49; *Nphs2*^*R140Q/-*^ n = 57) and (C) moderately increased mean arterial blood pressure (MABP) (control n = 25; *Nphs2*^*R140Q/-*^ n = 34; * p<0.05, ** p<0.005, *** p<0.001 vs. controls at the same time points). (D-F) *Nphs2*^*R140Q/-*^ mice display significant hypoalbuminemia, hypercholesterolemia and diminished creatinine clearance (columns represent 4–10 animals per group and time point). (G) Kaplan-Meier analysis of survival of 76 *Nphs2*^*R140Q/-*^ mice following induction vs. 30 healthy controls. Median survival time was 12 (95% CI: 11–13) weeks (p<0.01).

Proteinuria became detectable within few days (day 7: 3.48 ± 0.74 g/g creatinine in *Nphs2*^*R140Q/-*^ mice vs. 1.70 ± 0.16 g/g creatinine in healthy controls) and increased to a maximum 4–5 weeks after induction of *Nphs2*^*R140Q/-*^ using tamoxifen. Thereafter proteinuria decreased gradually but remained much elevated compared to controls until the end of the observation period (Fig 1A). Weight gain ceased in the proteinuric animals, leading to significantly lower body weight from week 6 onward (Fig 1B). Blood pressure moderately increased in *Nphs2*^*R140Q/-*^ animals compared to healthy controls (Fig 1C). Four weeks after induction *Nphs2*^*R140Q/-*^ animals displayed hypoalbuminemia (20.4 ± 1.9 g/l vs. 30.8 ± 1.7 g/l), hypercholesterolemia (345 ± 34 mg/dl vs. 76 ± 6.9 mg/dl) and diminished creatinine clearance (121 ± 21 μl/min vs. 171 ± 33 μl/min) compared to healthy controls (Fig 1D–1F). The median survival time of the *Nphs2*^*R140Q/-*^ animals was 12 (95% CI: 11–13) weeks (Fig 1G).

### Elevated R140Q *Nphs2* expression but loss of podocin protein abundance in *Nphs2*^*R140Q/-*^ mice

**Fig 2:**
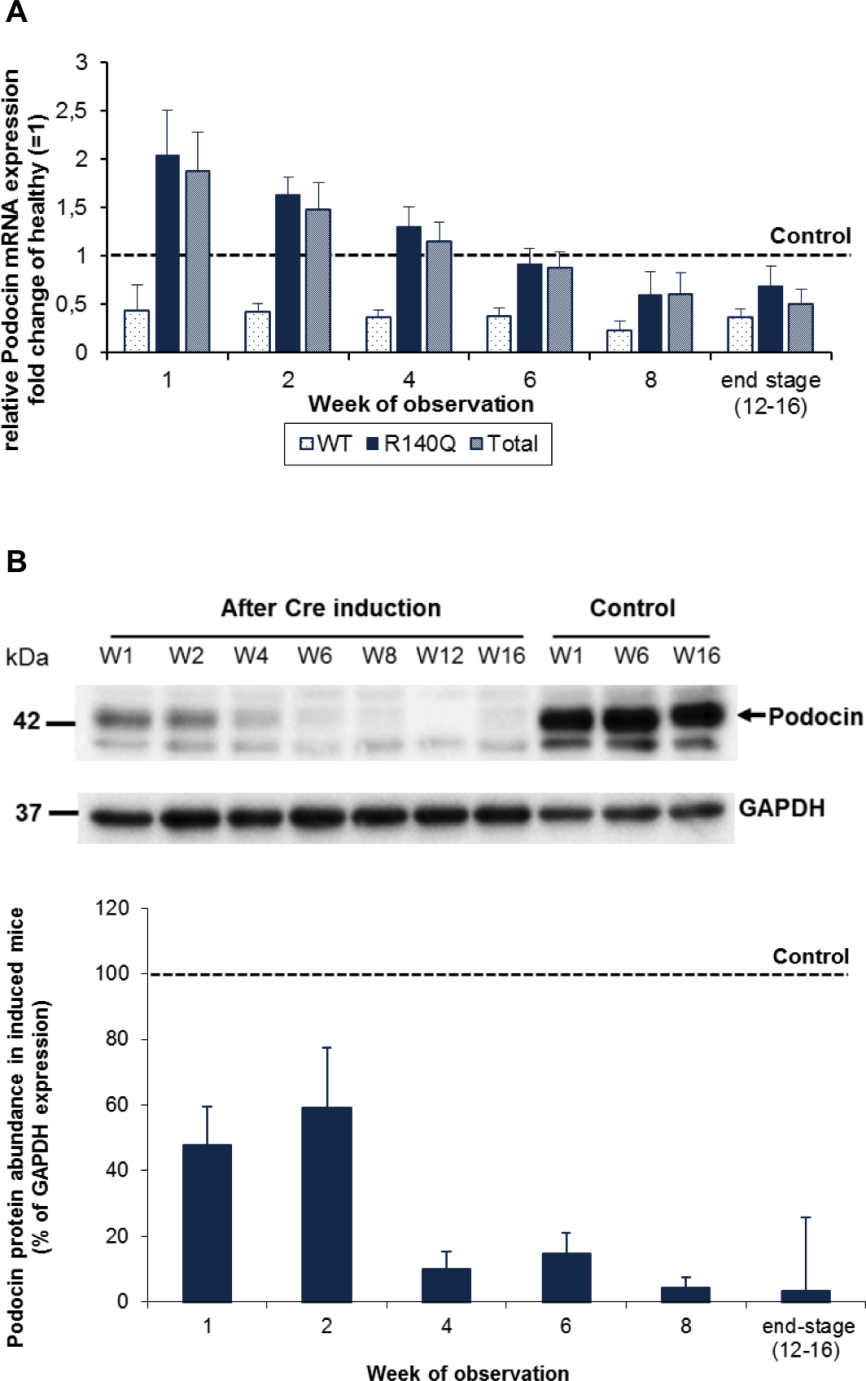
Whereas mRNA expression of mutant *Nphs2* is elevated, podocin protein abundance is diminished. (A) *Nphs2*^*R140Q/-*^ animals showed an elevated expression level of mutated podocin mRNA during the first four weeks following induction (analysis is based on 4–6 animals per group and time point) WT: wild type. (B) Western blot analysis of total kidney extracts showing partial podocin protein loss during first two weeks, subtotal loss after 4–6 weeks and complete loss at attainment of end-stage renal disease (week 12–16) (analysis is based on 4–6 animals per group and time point; p<0.05).

The expression of wild type (wt) podocin mRNA rapidly decreased after induction of the hemizygous state. Notably, R140Q podocin mRNA expression was elevated in *Nphs2*^*R140Q/-*^ animals during the first four weeks of observation compared to healthy controls (Fig 2A).

**Fig 3:**
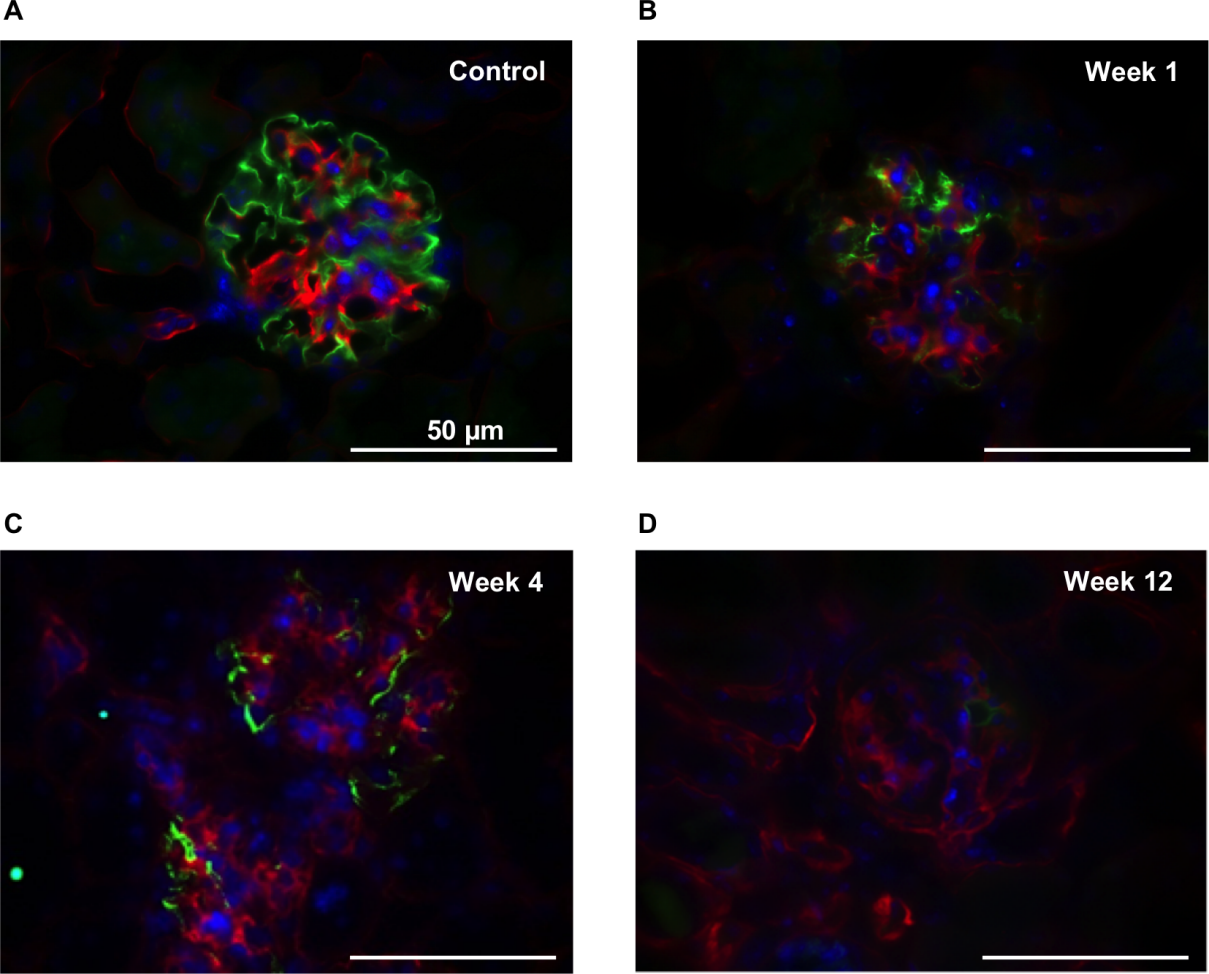
Progressive loss of glomerular podocin abundance in the course of disease. Podocin (green), nidogen (red) and nucleus (blue) staining of glomeruli of healthy and *Nphs2*^*R140Q/-*^ animals. (A) Normally expressed podocin in glomerulus of a healthy animal. (B) Partial podocin loss one week after the induction. (C) Immense podocin loss in *Nphs2*^*R140Q/-*^ animals four weeks after the induction. (D) Subtotal to total podocin loss at the end stage disease. Magnification, X640.

By contrast, podocin protein abundance was significantly reduced starting from week one (to 48% of that of healthy controls) and was almost completely abolished eight weeks after induction (to 4% of that of healthy controls) (Fig 2B). The loss of podocin in *Nphs2*^*R140Q/-*^ animals progressed to subtotal to total podocin loss at the end stage disease. These findings were confirmed by immunofluorescence studies (Fig 3).

### Mice develop FSGS and tubulointerstitial fibrosis

**Fig 4:**
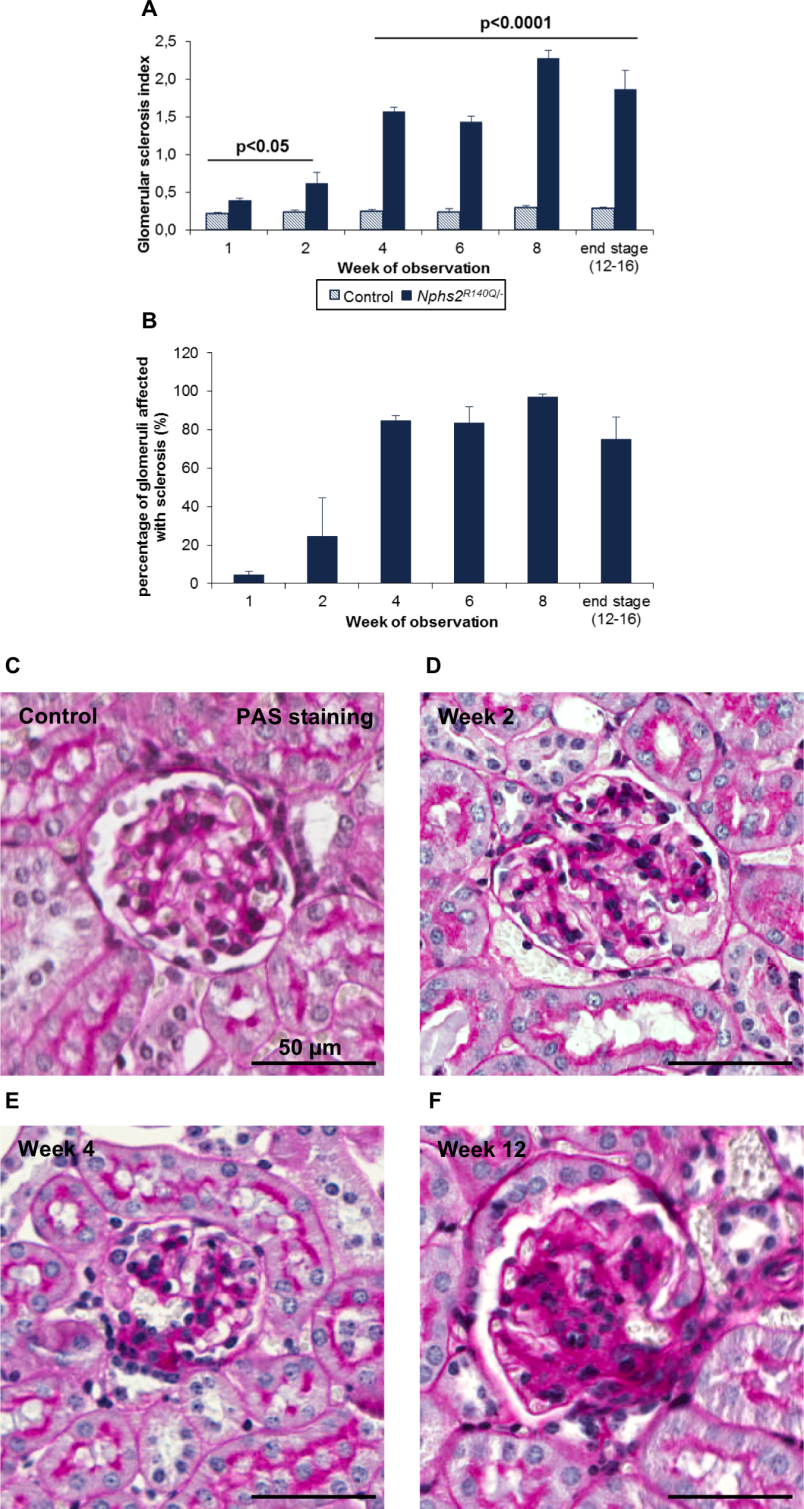
*Nphs2*^*R140Q/-*^ mice develop focal-segmental glomerulosclerosis (FSGS). (A) Glomerular sclerosis index (GSI) in healthy and *Nphs2*^*R140Q/-*^ mice. (B) Percentage of glomeruli affected by sclerosis in *Nphs2*^*R140Q/-*^ mice increased drastically over time (columns represent 4–10 animals per group and time point). (C-F) Evolution of glomerular lesions in induced animals. PAS staining; Magnification, X200.

**Fig 5:**
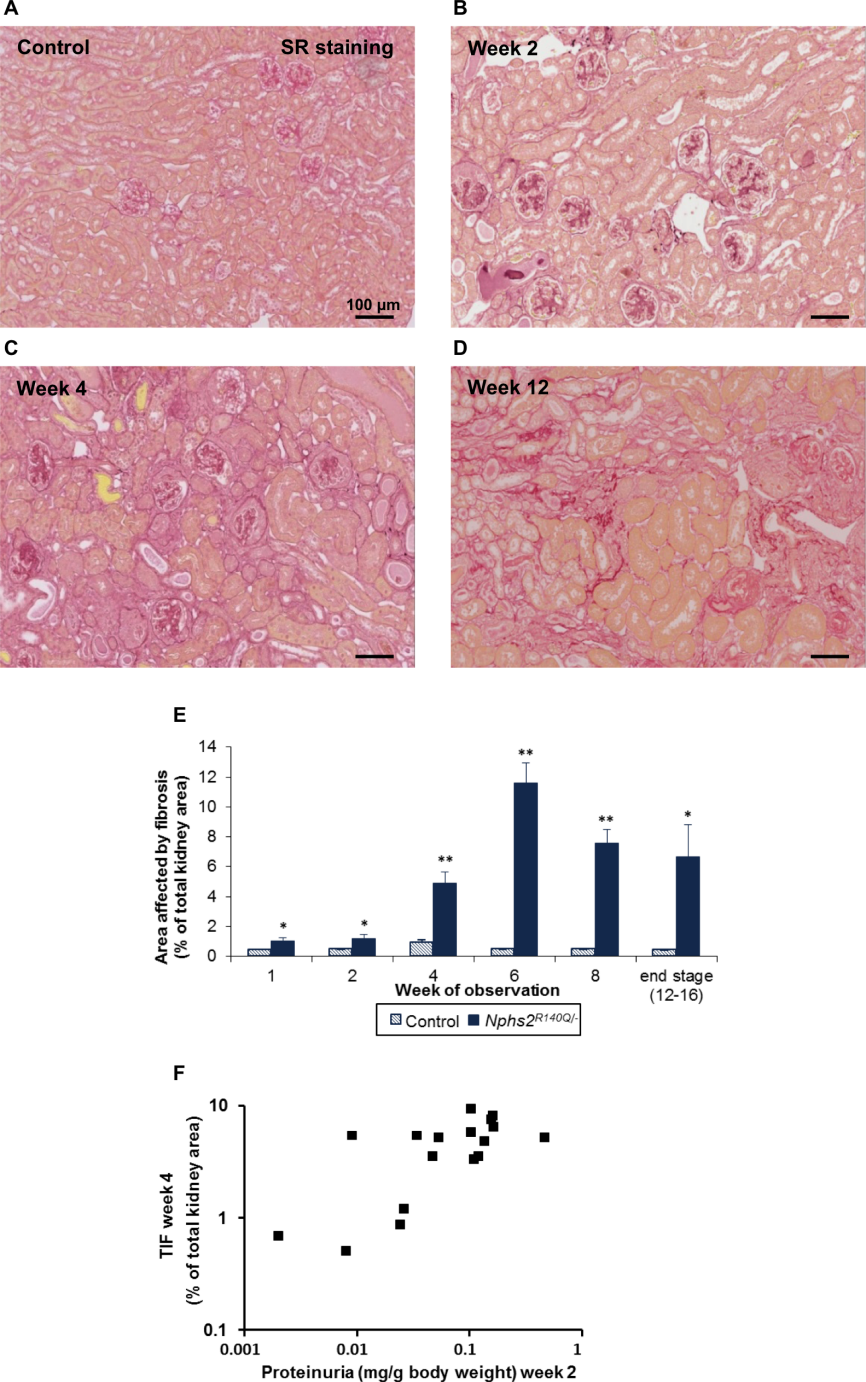
Podocin loss leads to renal damage. (A) control, (B) week 2, (C) week 4, (D) week 12. (E) Percentage of total kidney area affected by fibrosis in *Nphs2*^*R140Q/-*^ mice increased with observation time (columns represent 4–10 animals per group and time point). (F) Proteinuria 2 weeks after induction is correlated with the tubulointerstitial fibrosis score at week 4 (n = 17; p = 0.01). SR staining; Magnification, X150. * p<0.05, ** p<0.001.

The *Nphs2*^*R140Q/-*^ animals displayed significant early glomerular sclerosis from the first week after induction (glomerular sclerosis index (GSI) 0.39, p = 0.003). Subsequently the GSI progressively increased until week eight, when 100% of glomeruli were partially or globally sclerosed (Fig 4). In addition, the mice developed significant tubular atrophy and severe interstitial fibrosis, which were visible from the first week after induction and increased to 12% of the total kidney area in the course of disease until end-stage kidney disease was reached (Fig 5A–5E). Tubulointerstitial fibrosis at four weeks was quantitatively correlated with the degree of proteinuria two weeks after disease induction (p = 0.01; Fig 5F).

### *Nphs2*^*R140Q/-*^ mice show reduced podocyte number and podocyte foot process effacement

**Fig 6:**
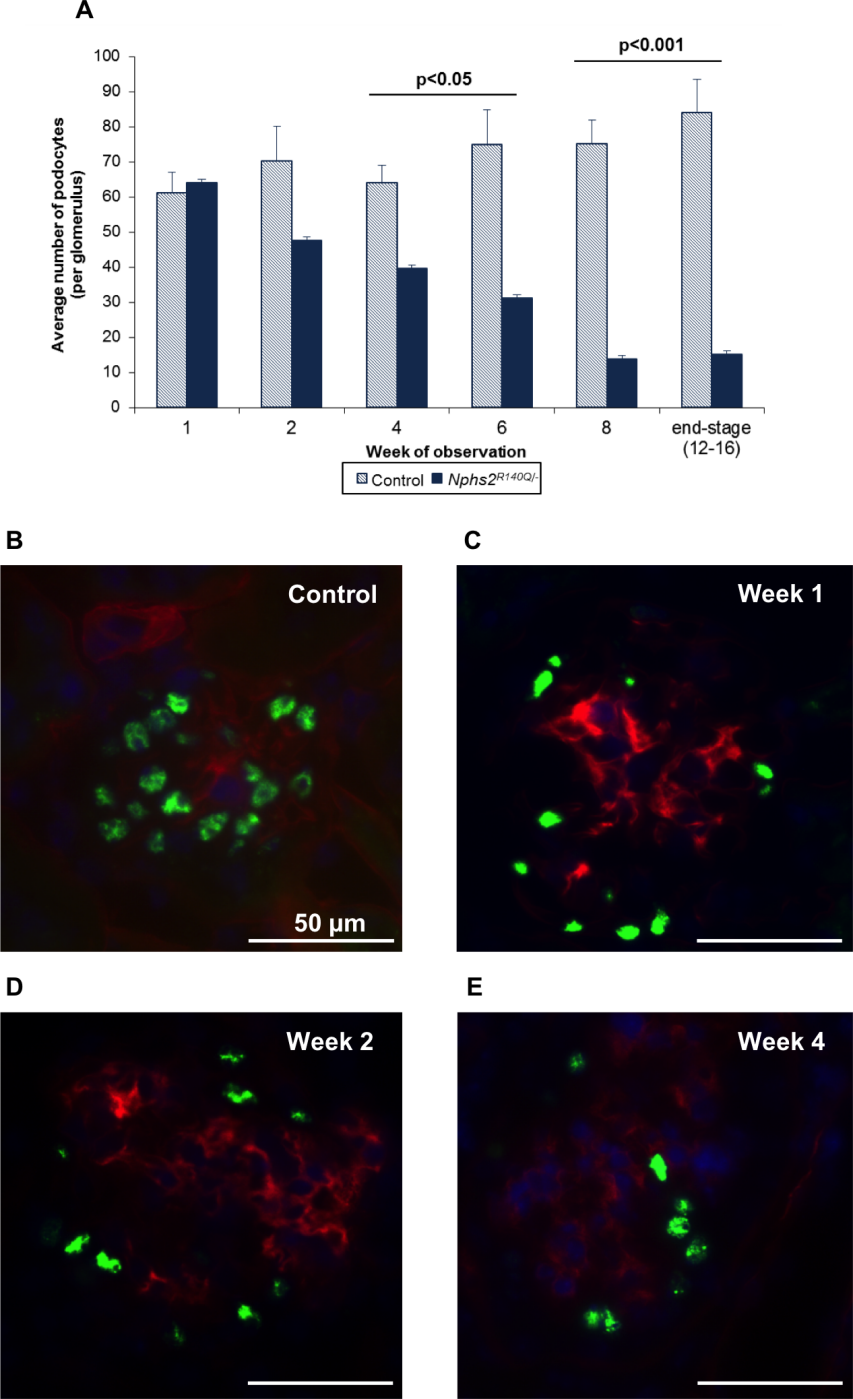
Podocyte loss in *Nphs2*^*R140Q/-*^ mice. (A) Number of podocytes reduced in induced mice with the course of the disease (columns represent 4–6 animals per group and time point). (B) Wt1 labelled podocytes in glomerulus in a healthy animal. Decreased Wt1 signal in *Nphs2*^*R140Q/-*^ animals 1 week (C), 2 weeks (D), and 4 weeks after induction (E). Wt1: green, nidogen: red, nucleus: blue. Magnification, X640.

**Fig 7:**
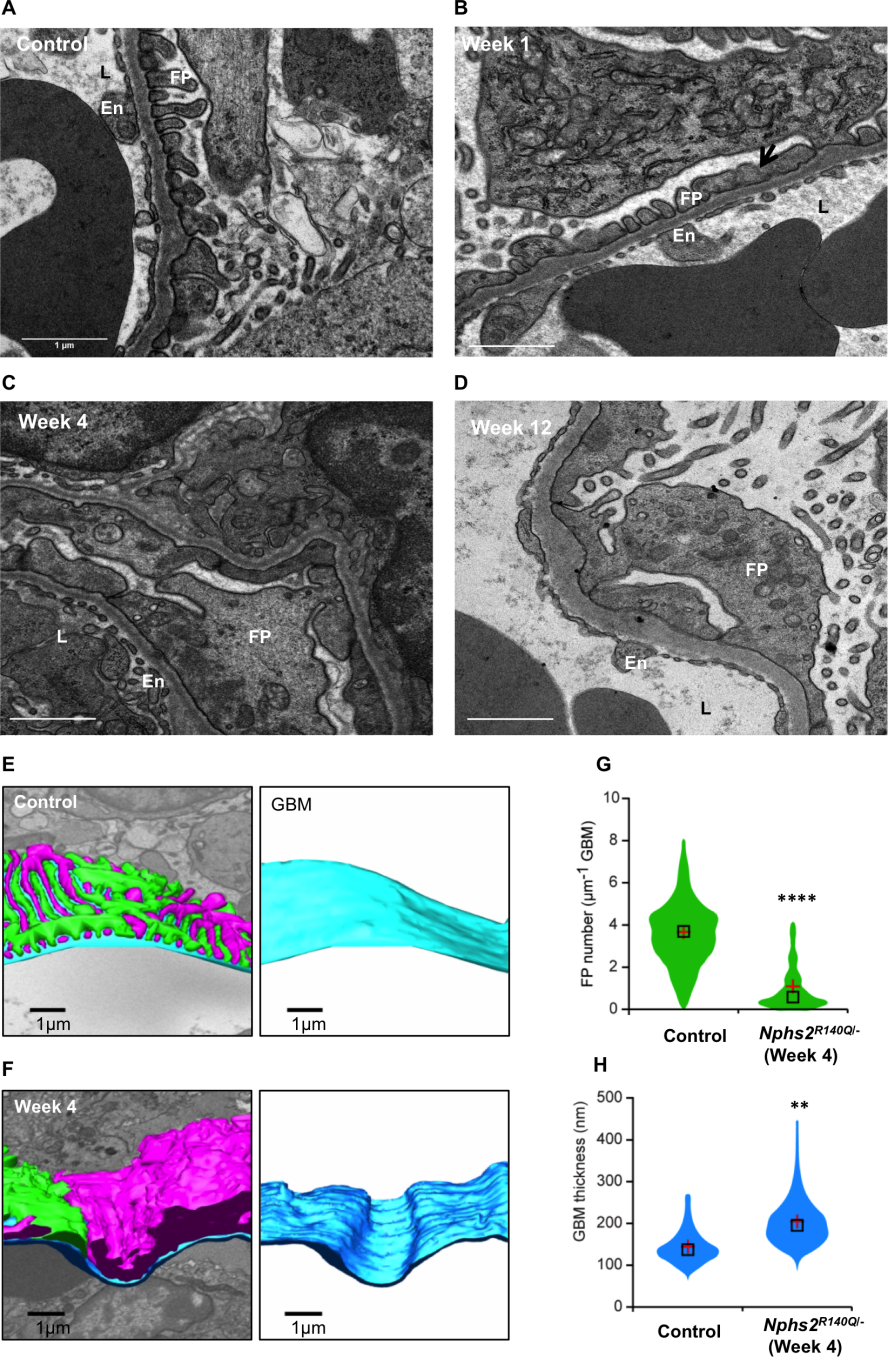
Podocyte foot process effacement in *Nphs2*^*R140Q/-*^ mice. (A) Ultrastructural studies showed regular foot processes (FP) in healthy control animals on the opposite side of endothelial cells (En) lining the capillary lumen (L). (B) Irregularly shaped or fused FPs in *Nphs2*^*R140Q/-*^ mice one week after induction (arrow). (C and D) Progression of focal changes to global fusion of FPs in *Nphs2*^*R140Q/-*^ animals over time (Magnification, X10000). 3D modelling of glomerular structure showed no GBM denudation in *Nphs2*^*R140Q/-*^ animal (F) compared to controls (E). Blue colour represents GBM, pink and green represent FPs of adjacent podocytes. Severely affected FP number and organization in *Nphs2*^*R140Q/-*^ animals (G). GBM thickening in *Nphs2*^*R140Q/-*^ animals (H) (G and H: analysis is based on 3 animals per group). ** p<0.01, **** p<0.0001.

The number of podocytes per glomerulus progressively diminished with time starting at the end of the second week after induction (68% of that of healthy controls) to 18% of that of healthy controls at 12–16 weeks (Fig 6A). Podocyte loss was also visualized by Wt1 labeling (Fig 6B–6E). Electron microscopy showed irregularly shaped or fused foot processes one week after induction (Fig 7A and 7B), progressing to effacement and global fusion at subsequent time points (Fig 7C and 7D). At variance to the previously described glomerular basement membrane (GBM) denudation as a mechanism of glomerular sclerosis [14], our *Nphs2*^*R140Q/-*^ mice did not display any area of GBM denudation in ultrastructural studies. 3D modelling of the glomerular structure revealed GBM thickening in induced animals relative to controls. Moreover, four weeks after induction the podocyte foot processes number per μm GBM was significantly decreased in addition to severe loss of foot process organization (Fig 7E–7H).

### miRNA studies

**Fig 8:**
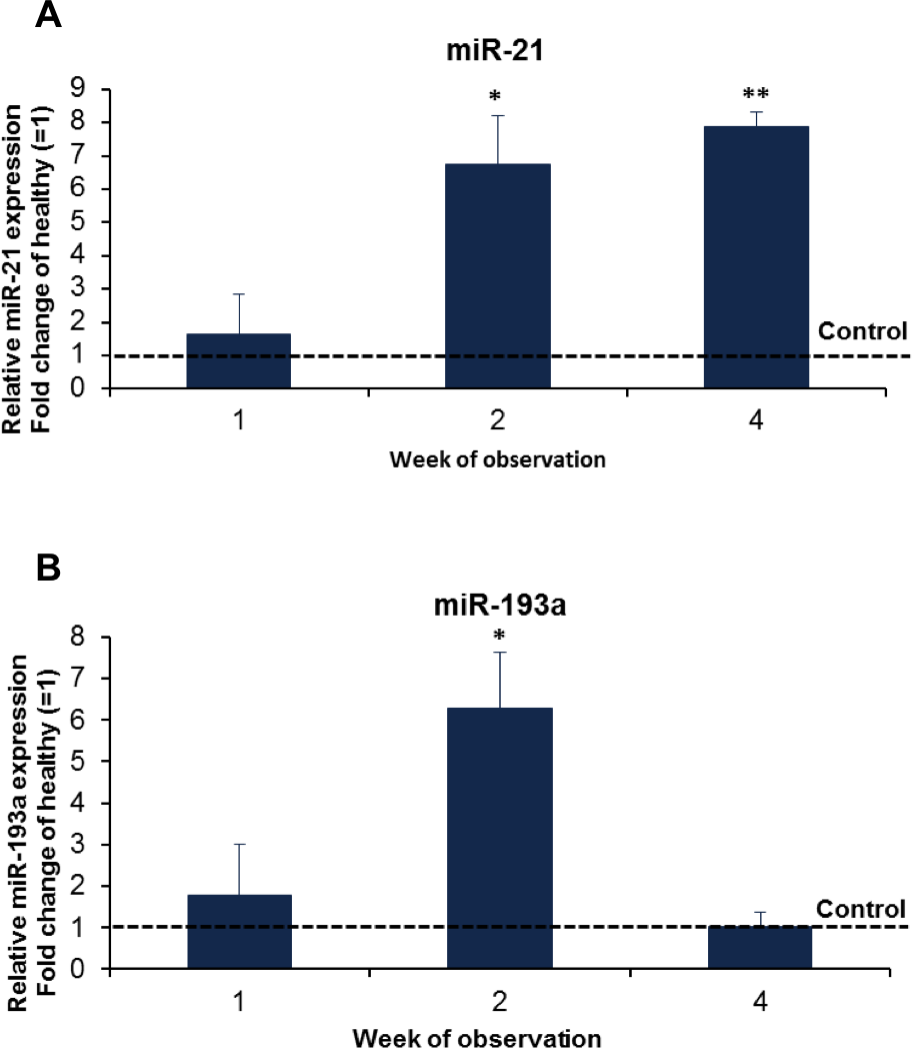
miRNA upregulation upon disease induction. Upregulation of microRNA 21 (A) and microRNA 193a (B) in *Nphs2*^*R140Q/-*^ animals (analysis is based on 4–6 animals per group and time point). * p<0.05, ** p<0.001.

To investigate possible post-transcriptional regulation of gene expression in our model, we searched for differences in the expression of miRNA-21 and miRNA-193a, previously described to be involved in chronic kidney failure [15–21]. In kidneys of *Nphs2*^*R140Q/-*^ and control animals one, two and four weeks after induction, miRNA21 expression persistently increased during the first four weeks of disease whereas miRNA-193a expression was increased by 100% within one week after induction and reached a peak after two weeks (Fig 8).

## Discussion

The induction of hemizygous R140Q podocin expression in our model resulted in a progressive glomerulopathy with a nephrotic phenotype gradually evolving to renal failure. The histopathological sequence of renal lesions includes foot process effacement, podocyte loss, glomerulosclerosis and tubulointerstitial fibrosis.

Previous mouse models of hereditary podocytopathies included deficiency or selective mutation of nephrin [22], podocin [10, 11], α-actinin 4 [23], *CD2AP* [24], laminin β2 [25–27], *TRPC6* [28] and α3 (IV) collagen [29–31] (Table 1).

**Table 1:** Time course of clinical phenotype in different hereditary podocytopathy mouse models.

	proteinuria	hypoalbuminemia	hyperlipidemia	hypertension	FSGS	FP effacement	renal failure	average survival time	remarks
***Nephrin* TRAP mice**	soon after birth				fibrotic glomeruli at birth	at birth	Prenatal or immediately after birth	P1	
***laminin beta2* mutant mice**	1–3 wk of age				3 wk of age	P15		1 mo	edematous near the time of death; GBM thickening
***NPHS2* KO mice**	present at birth				no FSGS	E16.5	within 5 wk after birth	7–23 days depends on genetic background	hemorrhage, DMS, endothelial lesions
***Nphs2***^***R140Q/R140Q***^ **knockin mice**	present at P4				P32	present at P10	within the first month of life	4–14 days of age depends on genetic background	hemorrhage, DMS, endothelial lesions
**conditional *Nphs2* knockout mice**	8–13 days after induction		4 wk after induction	4 wk after induction	4 wk after induction	1–2 wk after induction	6–9 wk after induction	11 wk after induction	30% with glomerular pseudocrescent
**conditional *Nphs2* R140Q knockin mice**	few days after induction	4 wk after induction	4 wk after induction	4 wk after induction	2 wk after induction	1–2 wk after induction	8–12 wk after induction	12 wk after induction	GBM thickening
***Trpc6* mutant mice**	5–9 mo of age; incomplete penetrant phenotype				5–9 mo of age	3 mo of age			only male animals; GBM thickening
***alpha Actinin* mutant mice**	1–3 wk of age; incomplete penetrant phenotype			10 wk of age	10 wk of age	10 wk of age			no change in GBM thickness
***CD2AP* deficient mice**	2 wk of age	4 wk of age			4 wk of age	1 wk of age	6–7 wk of age	6–7 wk of age	
**Alport mouse model**	P40				P40		P65	3 mo of age	

DMS: diffuse mesangial sclerosis; E: embryonic; GBM: glomerular basement membrane; mo: month; P: postnatal; wk: week

Most of these models are characterized either by severe renal developmental defects and congenital nephrotic syndrome leading to early demise [10, 11, 22, 25–27] or by incomplete penetrance [23, 28]. The conditional inactivation of normal protein expression achieved by use of an inducible CreLox system allows inducing and describing comprehensively a uniform renal phenotype established at postnatal age, well after completion of nephrogenesis. In our model, proteinuria appeared within days after induction of the hemizygous knockin state and peaked after four weeks. Hypoalbuminemia and hyperlipidemia, the other key biochemical features of nephrotic syndrome, manifested within two weeks of disease induction. Proteinuria gradually decreased in the second and third month as the animals developed progressive FSGS, hypertension and renal failure. Death from uremia occurred mainly between week 6 and 16, with an average survival time of 11 weeks.

The observed phenotype closely resembles that of the inducible podocin knockout mouse model previously developed by the group of C. Antignac, with similar time courses of proteinuria, glomerulosclerosis and death in uremia [12]. However, the model presented here provides a better molecular recapitulation of human disease as complete podocin deficiency rarely occurs in humans. Furthermore, the production of a podocin protein with defective folding due to a single amino acid exchange and consecutive retention in the ER renders the model suitable for *in vivo* screening of candidate chaperone compounds that would stimulate protein trafficking to the membrane.

With regards to the timing and extent of histopathological and ultrastructural changes, it is worth noting that at the earliest stage of disease mild proteinuria was observed in the presence of still almost normal EM findings (showing some unincisive irregular FPs), whereas the progression to higher grade proteinuria was associated with pronounced foot process fusion and effacement (Fig 7). These findings are in keeping with previous reports of hereditary podocytopathy models including the inducible podocin kockout model [5, 12, 32]. No invasion of GBM by podocytes was observed as has been reported for collagen IV nephropathy and other models of glomerular disease [33].

FSGS is the typical histopathological lesion found in renal biopsies of patients with steroid resistant nephrotic syndrome (SRNS) due to *NPHS2* mutations [2, 34, 35]. The *Nphs2*^*R140Q/-*^ mice demonstrated very similar histomorphology starting with segmental glomerular scarring in individual glomeruli that progressed gradually to almost all glomeruli, leading to terminal renal failure. Several FSGS subtypes including the collapsing, glomerular tip lesion and crescent variants were observed along with diffuse foot process effacement and podocyte loss. These lesions might occur secondary to podocyte detachment [36, 37] or result from altered expression of genes encoding important structural or functional components of the podocytes [36].

Toxin-induced podocyte depletion studies in a transgenic rat model revealed a quantitative correlation of glomerulosclerosis with the extent of podocyte loss [38]. Also in the model presented here, the development and progression of glomerulosclerosis is likely to result from gradual podocyte depletion. In support of this notion, podocyte loss correlated with the significant increase of the glomerular sclerosis index, which was noted four weeks after induction. While podocyte loss is believed to be essential for developing glomerulosclerosis, the mechanisms leading to podocyte detachment remains elusive. Specifically, it is not even clear whether podocytes detach because they die or due to loss of adhesion [36]. Podocyte loss in this model is compatible with either mechanism since intact podocin is essential both for podocyte survival and for the maintenance of foot process structure and function [39]. Furthermore, although no GBM denudation could be detected in our model, massive podocyte hypertrophy observed in our animals (e.g. Fig 7D) could promote podocyte stress leading to podocyte loss and subsequent development of glomerulosclerosis [40, 41]. The percentage of sclerotic glomeruli, as well as the area affected by sclerosis in each glomerulus, increased with time in *Nphs2*^*R140Q/-*^ animals, a process presumably enhanced by increasing hyperfiltration stress to podocytes in remaining glomeruli once a critical mass of nephrons was obliterated.

Tubulointerstitial fibrosis was visible from the end of the first week after disease induction and was enhanced after four weeks (Fig 5). The degree of tubulointerstitial fibrosis was correlated with the preceding degree of proteinuria after disease induction. Ample experimental evidence supports that nephrotic range albuminuria is toxic to tubular epithelial cells and a potent cause of tubulointerstitial inflammation and fibrosis [42]. In addition, it has been hypothesized that ‘misdirected filtration’ with diffusion of ultrafiltrate into the peritubular space may occur in segmental glomerulosclerosis following synechial attachment of the GBM to Bowman’s capsule [43].

On the molecular level, we observed a gradual loss of podocin protein (both by western blotting and immunofluorescence staining) following induction of the hemizygous knockin state. A reduction of podocin abundance by more than 50% was observed within one week following induction. Due to the inactivation of the floxed wt podocin allele in *Nphs2* ^*R140Q/-*^ animals the expression of wt podocin mRNA decreased, however R140Q podocin mRNA expression was elevated during the first four weeks after induction. Significant upregulation of R140Q podocin mRNA was previously showed in the R140Q podocin knockin mouse model [10]. The diminished podocin protein abundance in face of elevated mutated *Nphs2* gene expression is in keeping with intracellular retention and increased proteasomal degradation of mutant protein as previously demonstrated *in vitro* for the R138Q mutation [44, 45]. When glomerulosclerosis was fully established after four weeks, podocin abundance had dropped to less than 10% of that of controls. At that time the number of podocytes per glomerulus had only decreased by one third of that of healthy controls and R140Q podocin mRNA expression was still elevated, indicating that podocin protein loss at this stage of the disease was a specific consequence of the hemizygous mutated state with enhanced intracellular degradation rather than a consequence of early global podocyte depletion.

**Fig 9:**
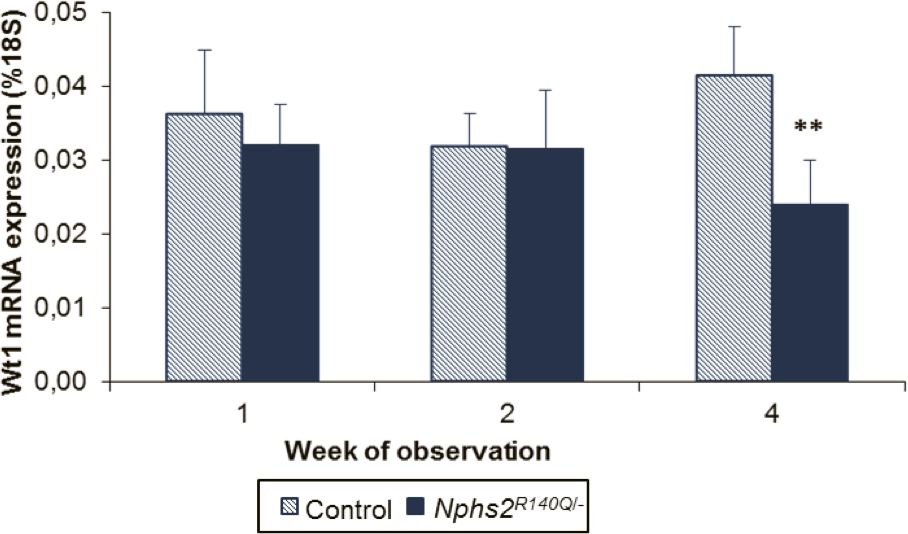
*Wt1* regulation in induced animals. *Wt1* expression is significantly reduced in *Nphs2*^*R140Q/-*^ animals 4 weeks after disease induction (columns represent 5–7 animals per group and time point). **p = 0.0002.

In order to elucidate key molecular responses to the podocin mutated state, we chose to study the expression of miR-193a and miR-21 based on a review of the recent literature. miR-193a was found highly upregulated in glomeruli isolated from FSGS patients and it has been shown that its overexpression in an inducible mouse model led to focal and later global glomerulosclerosis with broad capsular adhesion [15, 16]. We observed an early and transient upregulation of miR-193a in our model, supporting the notion that this miRNA is an important player in the sequence of molecular events leading to FSGS in hereditary podocytopathies caused by functional deficiency of a single podocyte protein. The major target of miR-193a is *WT1*, which plays an essential role in the development and maintenance of podocytes [16, 46, 47]. Repression of WT1 upon upregulation of miR-193a initiates a cascade of events resulting in structural destabilization and disrupted function of podocytes, leading to progressive FSGS [16]. Intriguingly, we found that upregulation of miR-193a two weeks after disease induction was followed by a reduction in *Wt1* expression in week 4 (Fig 9).

The function of miR-21 in animal models of kidney injury is controversial. In some models of kidney disease upregulation of miR-21 promotes kidney injury [17–19], whereas other studies suggest that increased miR-21 expression might rather activate protective molecular programs that would attenuate tubulointerstitial and glomerular injury [20, 21]. Our finding that miR-21 is upregulated in *Nphs2* ^*R140Q/-*^ animals compared with controls is consistent with a function of miR-21 in promoting glomerular injury.

In conclusion, we have generated an inducible mouse model of podocin nephropathy with a disease course and histopathological features closely resembling the human disorder. The inducible *Nphs2* ^*R140Q/-*^knockin mouse is the ideal model to test the efficacy of antiproteinuric therapies including novel molecular approaches targeting the underlying molecular defect.

## Materials and methods

### Generation of Mice

C57BL/6 mouse lines transgenic for *Cre*^*+/-*^ [13], *Nphs2*^*+/-*^ and *Nphs2*^*lox2/lox2*^ and *Nphs2*^*+/R140Q*^ were previously established in collaboration with the Mouse Clinical Institute (Institut Clinique de la Souris, Illkirch, France) and described in Mollet et al., 2009 and Philippe et al., 2008, respectively. The two lines *Cre*^*+/-*^, *Nphs2*^*+/-*^ and *Nphs2*^*lox2/lox2*^ were crossed and offspring mice with *Nphs2*^*lox2/+*,^ *Cre*^*+/-*^ and *Nphs2*^*lox2/-*,^ *Cre*^*+/-*^ genotypes were subsequently used for further matings. *Nphs2*^*lox2/lox2*^, *Cre*^*+/+*^ mice, resulting from mating between *Nphs2*^*lox2/+*^, *Cre*^*+/-*^ and *Nphs2*^*lox2/-*^, *Cre*^*+/-*^ mice, were then crossed with the *Nphs2*^*+/R140Q*^ mice. The resulting *Nphs2*^*lox2/R140Q*^, *Cre*^*+/-*^ mice were used for the final experiments.

All animal experiments were performed in compliance with EU regulations for animal experimentation. The project was approved by the Government of Baden-Württemberg, Regierungspräsidium Karlsruhe, Germany under Aktenzeichen 35–9185.81/G-191/09 and 35–9185.81/G-181/15.

### Experimental design for phenotypic characterization

Hemizygosity for the R140Q allele was induced in six weeks old *Nphs2*^*lox2/R140Q*^, *Cre*^*+/-*^ mice by intraperitoneal administration of tamoxifen (33 mg/kg/day for 5 days; Sigma-Aldrich, St.Louis, MO), dissolved in sunflower oil (Sigma-Aldrich, St.Louis, MO). The resulting mice were named *Nphs2* ^*R140Q/-*^ in the manuscript. Control mice with the same genotype (*Nphs2*^*lox2/R140Q*^, *Cre*^*+/-*^) received the equivalent volume of sunflower oil without tamoxifen. Mice were maintained in a pathogen-free environment (S1 Table) of Interfaculty Biomedical Facility (IBF) (Clinical experimental area- KEB), Ruprecht-Karls-University Heidelberg, where their health and behaviour were controlled daily by skilled animal takers. All experiments were conducted in accordance with the German Law on Animal Welfare (TierSchG), paragraph § 8 –animal experiments—from 7 June 2006 (BGBI. I S. 1313). All research staff handling with animals was trained in accordance with the recommendations of the GV-SOLAS (Society of laboratory animal science) and the FELASA (Federation of European Laboratory Animal Science Associations) in a class B course.

The onset and course of proteinuria was monitored by weekly collection of 24-hour urine in metabolic cages (Tecniplast, Hohenpeißenberg, Germany). Proteinuria was monitored weekly until the time of sacrificing.

Starting from the first week after tamoxifen administration, the animals were accustomed to tail-cuff plethysmography according to the manufacturer’s recommendations (Coda—Kent Scientific Corporation). Thereafter, blood pressure measurements (25 cycles per session per mouse) were obtained once weekly by tail-occlusion plethysmography. The median values of systolic, diastolic and mean blood pressure of accepted cycles were taken as representative for the session.

At least four control and four induced mice were sacrificed at weeks 1, 2, 4, 6, 8, 12 and 16 after tamoxifen administration or followed open end for max. 35 weeks for survival studies. In case of anorexia of about 50% or cachexia over 10%, as specific endpoint criteria, the affected animal was euthanized immediately. Only 16 animals died spontaneously during the whole observation time. At defined experimental time points animals were deeply anesthetized with CO2 and blood was obtained by cardiac puncture. The sacrificed mice were then perfused with 10 ml 0.9% NaCl solution. Both kidneys were excised and decapsulated and samples were collected for RNA and protein isolation, histopathological analysis, immunofluorescence and electron microscopy. Urine protein concentration was measured according to the Bradford method. Blood urea, serum albumin, creatinine and cholesterol, and urinary creatinine were measured using standard laboratory methods.

### Real time rtPCR

Approximately 1 μg of total RNA obtained from kidney tissue blocks using the RNeasy Mini Kit (QIAGEN, Hilden, Germany) was reverse transcribed with oligo (dT)/random hexamer primers (10:1). Real time reverse transcription polymerase chain reaction (RT-PCR) was performed with the StepOne Plus Real Time PCR system (Applied Biosystems, Foster City, CA, USA) with specific primers for 18 S (forward: 5'-AGTTGGTGGAGCGATTTGTC- 3'; reverse: 5'-CGGACATCTAAGGGCATCAC- 3'), *Nphs2 WT* (forward: 5'-GTCCTCGCCTCCCTGATCTT-3'; reverse: 5'-AGGAAGCAGATGTCCCAGTC-3'), *Nphs2 R140Q* (forward: 5'-GTCCTCGCCTCCCTGATCTT-3'; reverse: 5'-AGGAAGCAGATGTCCCAGCT-3'), *Nphs2 total* (forward: 5'-TTGATCTCCGTCTCCAGACCTT-3'; reverse: 5'-TCCATGCGGTAGTAGCAGACA- 3'), *Wt1* (forward: 5'-GAAATGGACAGAAGGGCAGA-3'; reverse: 5'-GACCCCGTGGGTGTGTATT-3'), and Universal Mastermix (Applied Biosystems) with SYBR green to detect PCR products at the end of each amplification step. Serial dilutions of an arbitrary cDNA pool were used to establish a standard curve. Relative quantities of RNA levels were determined, accounting for amplification efficacy by the software provided with the PCR system. Messenger RNA levels were normalized to corresponding 18S quantities, determined within the same run.

The analysis of miRNA-21 (5'- UAGCUUAUCAGACUGAUGUUGA-3') and miRNA-193a (5'- AACUGGCCUACAAAGUCCCAGU-3') was performed by qPCR by CBC (Comprehensive Biomarker Center GmbH, Heidelberg, Germany). Delta CT values were normalized to endogenous snoRNA 202 (NCBI Accession: AF357327) and snoRNA 234 (NCBI Accession: AF357329).

### Western immunoblotting

Snap-frozen kidney tissues were homogenized on ice with CHAPS buffer containing protease inhibitors (10 μl CHAPS buffer per mg tissue). Samples were then vortexed and centrifuged (4000 g) for 35 min at 4°C. Protein concentration of the supernatant was measured according to Bradford method and 30 μg of total protein were denatured in 4× sample buffer separated by SDS polyacrylamide gel electrophoresis (12%, SDS-PAGE) and transferred to a PVDF Transfer membrane (Millipore, Billerica, MA, USA). The membranes was blocked in 5% milk and 3% bovine serum albumin (BSA) for 1 h at room temperature, then incubated with primary rabbit anti-podocin antibody (Sigma) overnight at 4°C (1:1000), followed by incubation with the second antibody (HRP-anti-rabbit, 1:3000; Cell Signaling, Danvers, MA, USA). The membrane was visualized using chemiluminescent detection. Equal protein loading was confirmed by reprobing the membrane for GAPDH (1:20000) (Meridian Life Sciences, Memphis, USA). Signal intensity was evaluated densitometrically (Quantity one software, Bio-Rad, Hercules, CA).

### Histopathological analysis

Kidney tissues fixed in 4% buffered formalin were paraffin-embedded and 3 μm thick sections were stained with Masson-Trichrome for qualitative and Sirius Red for quantitative analysis of tissue fibrosis, and with periodic acid-Schiff (PAS) to determine the degree of sclerosis within the glomerular tuft.

Samples stained with Sirius Red were scanned using Aperio Spectrum software (Aperio Technologies). Images were captured using Aperio ImageScope software at total magnification of 300. The percentage of sirius red–stained tubulointerstitial area was measured in 30 randomly sampled fields per kidney using Image-Pro Plus software (Media Cybernetics, Silver Spring, MD) as described previously [48].

The degree of sclerosis within the glomerular tuft as a parameter of renal disease progression was determined in a blinded manner on PAS-stained paraffin sections using the semiquantitative scoring system [49]. Images of scanned sections were captured at total magnification of 300. For each animal the glomerular sclerosis index (GSI) was derived as the mean of 50 glomeruli. The glomerular score for individual glomeruli was: grade 0—normal glomerulus; grade 1—mesangial proliferation/sclerosis involving less than 25% of the glomerular tuft; grade 2—sclerosis involving more than 25%, but less than 50% of the glomerular tuft; grade 3—sclerosis involving more than 50%, but less than 75% of the glomerular tuft; grade 4—diffuse glomerulosclerosis with total tuft obliteration. The fractional sclerosed area within the glomerular tuft was determined using Image Pro Plus software (Media Cybernetics, Silver Spring, MD).

The number of podocytes per glomerulus was determined according to the Animal Models of Diabetic Complications Consortium protocol (www.diacomp.org). Samples were treated with Wt1 antibody from LifeSpan Biosciences, Seattle, WA.

### Immunofluorescence microscopy

Tissues frozen in Tissue-Tek Gel (Sakura, Alphen aan den Rijn, the Netherlands) were cut in 5 μm sections, fixed in acetone/methanol solution (1:1), washed with PBS and incubated with DCS antibody dilution buffer (Innovative Diagnostik-Systeme, Hamburg, Germany) overnight at 4°C. Samples were then incubated with primary rat anti-mouse Nidogen antibody (1:6000) (Millipore) to stain the basal membranes for 1 hour at room temperature, followed by washing and treating with donkey anti-rat antibody (DyLight 549 red, Dianova, Hamburg, Germany) diluted 1:2000 for 1 hour at room temperature. After washing steps, samples were blocked again in DCS for 1 hour at room temperature. After just tipping the blocking solution away the primary rabbit anti mouse podocin antibody diluted (1:1000) (Sigma) or the primary rabbit anti mouse Wt1 antibody diluted (1:400) (LifeSpanBiosciences, Seattle, WA, USA) was added and incubated overnight at 4°C or 1 hour at room temperature, respectively. After washing the secondary donkey anti rabbit antibody (DyLight 488 green, Dianova) (1:1000 for Podocin and 1:250 for Wt1) was added (1 hour, room temperature). Nuclei were labelled using bisbenzimide (diluted 1:1000 in water) (Serva, Heidelberg, Germany). Slides were examined using a Leica DMI 4000 B microscope (Leica, Deerfield, IL, USA).

### Transmission electron microscopy

Renal tissue samples (1 mm^3^) were stored in EM fixation solution (phosphate buffer containing 1.5% Glutaraldehyde und 1.5% Paraformaldehyde) at 4°C until embedding. Following embedding with Epon [50] and subsequent polymerisation, ultra-thin sections were obtained using diamond knives (Diatome®, Hatfield, PA or Drukker®, Drukker International BV, the Netherlands) with a Leica® UltraCut microtome (Leica, Deerfield, IL, USA). The samples were then stained with 2% aqueous uranyl acetate and lead citrate and observed under a ZEISS EM 10A transmission electron microscope (Carl Zeiss, Oberkochen, Germany).

### Serial block face-scanning electron microscopy

Kidney samples were prepared for SBF-SEM as described previously [51]. In brief, mouse kidneys were cut into 1 mm cubes and fixed in situ by using 2% (wt/vol) glutaraldehyde (Agar Scientific, UK) in 0.1 M cacodylate buffer (pH 7.2); stained in 1% (wt/vol) osmium tetroxide, 1.5% (wt/vol) potassium ferrocyanide in 0.1 M cacodylate buffer, followed by 1% (wt/vol) thiocarbohydrazide. After washing, more osmium was added by staining in 1% (wt/vol) osmium tetroxide, and soaked in 1% (wt/vol) uranyl acetate overnight. The final staining step incubation was performed at 60°C with lead aspartate pH 5.5 for one hour. Samples were dehydrated in ethanol and infiltrated in TAAB 812 hard resin. Tissue was mounted onto an aluminium cryo pin (Electron Microscopy Sciences, cat. no. 70446) using cyanoacrylate glue and all block surfaces trimmed at 90°C using a glass knife or diamond trimming tool (Diatome). A gold coating was applied to the block to create a conductive surface. The block was placed in the Quanta 250 FEG (FEI Company) + Gatan 3view system and a 41 μm × 41 μm field of view was chosen and imaged by using a 4096 × 4096 scan, which gave an approximate pixel size of 10 nm. The section thickness was set to 50 nm in the Z (cutting) direction.

### Quantitative ultrastructure analysis

Different blocks from the same sample used for SBF-SEM (Serial block face-scanning electron microscopy) were used to quantify the ultrastructure by means of TEM (Transmission Electron Microscopy). Tissue blocks were removed from their moulds and sectioned (70–80 nm thickness). Sections were examined using a FEI Tecnai 12G2 Biotwin transmission electron microscope. Magnification varied from 145X to 6800X. n = 6 glomeruli were imaged. Images were saved as original.dm3 files and.tiff files. The dm3 files were opened using Fiji/ImageJ software version 2.0.0-rc-29/1.49r. Collectively 60 randomly selected points of the GBM across 50.dm3 images were measured to assess GBM thickness. The straight-line option was used to draw across the width of the GBM at the chosen point >Analyse > Measure. To quantify podocyte FP number 50 randomly selected areas of GBM 5 μm in length were measured as above and FP number counted manually. All measurements were imported into GraphPad Prism software version 5.0 for interpretation and graphical representation.

### Statistical analysis

All data are presented as means ± SEM and compared using t test for independent samples. P < 0.05 was considered statistically significant.

## Supporting information

S1 Table Mouse housing in the clinical experimental area (KEB). (DOCX)
