# Supplementary material for: An inducible mouse model of podocin-mutation-related nephrotic syndrome
Source: PLoS One. 2017 Oct 19;12(10):e0186574. doi: 10.1371/journal.pone.0186574 (PMC5648285; doi:10.1371/journal.pone.0186574)
Supplement: S1 Table — (DOCX) [file pone.0186574.s001.docx]

**S1 Table. Mouse housing in the clinical experimental area (KEB).**

| **Housing** | **Mouse** |
| --- | --- |
| Type of facility | SPF |
| Cage type | II, conventional |
| Bedding | ABBEDD LT-E-001 |
| Number of cage companions | see recommendations GV-SOLAS |
| Humidity | 50-60% |
| Light-dark cycle | 12-12 |
| Temperature | 22°C +/- 2°C |
| Type of food | LasVendi Rod 16 or Rod 18 |
| Access to food | ad libitum |
| Environmental enrichment | yes, tissue, nestlets |
